# Identifying the Added Value of Virtual Reality for Treatment in Forensic Mental Health: A Scenario-Based, Qualitative Approach

**DOI:** 10.3389/fpsyg.2019.00406

**Published:** 2019-02-27

**Authors:** Hanneke Kip, Saskia M. Kelders, Kirby Weerink, Ankie Kuiper, Ines Brüninghoff, Yvonne H. A. Bouman, Dirk Dijkslag, Lisette J. E. W. C. van Gemert-Pijnen

**Affiliations:** ^1^Centre for eHealth and Wellbeing Research, Department of Psychology, Health and Technology, University of Twente, Enschede, Netherlands; ^2^Department of Research, Stichting Transfore, Deventer, Netherlands; ^3^Optentia Research Focus Area, North-West University, Vanderbijlpark, South Africa

**Keywords:** virtual reality, forensic mental health, psychological treatment, delinquent behavior, contextual inquiry

## Abstract

**Background:** Although literature and practice underline the potential of virtual reality (VR) for forensic mental healthcare, studies that explore why and in what way VR can be of added value for treatment of forensic psychiatric patients is lacking.

**Goals:** This study aimed to identify (1) points of improvements in existing forensic mental health treatment of in- and outpatients, (2) possible ways of using VR that can improve current treatment, and (3) positive and negative aspects of the use of VR for the current treatment according to patients and therapists.

**Methods:** Two scenario-based methods were used. First, semi-structured interviews were conducted with eight therapists and three patients to elicit scenarios from them. Based on these results, six scenarios about possibilities for using VR in treatment were created and presented to 89 therapists and 19 patients in an online questionnaire. The qualitative data from both methods were coded independently by two researchers, using the method of constant comparison.

**Results:** In the interviews, six main codes with accompanying sub codes emerged. Ideas for improvement of treatment were grouped around the unique characteristics of the forensic setting, characteristics of the complex patient population, and characteristics of the type of treatment. For possibilities of VR, main codes were skills training with interaction, observation of situations or stimuli without interaction, and creating insight for others into the patient. The questionnaire resulted in a broad range of insights into potential positive and negative aspects of VR related to the current treatment, the patient, the content of a VR application, and practical matters.

**Conclusion:** VR offers a broad range of possibilities for forensic mental health. Examples are offering training of behavioral and cognitive skills in a realistic context to bridge the gap between a therapy room and the real world, increasing treatment motivation, being able to adapt a VR application to individual patients, and providing therapists with new insights into a patient. These findings can be used to ground the development of new VR applications. Nevertheless, we should remain critical of when in the treatment process and for whom VR could be of added value.

## Introduction

Virtual reality (VR) has been rapidly gaining ground in mental health research and practice, and evidence so far has warmed many researchers and clinicians up to VR’s potential in improving treatment. In VR, patients can enter computer-generated environments, which substitute real-world sensory visual and auditory perceptions with virtual ones (Freeman et al., 2017). Ideally, this will elicit a sense of presence, which is the illusion of actually being in a place, while one is physically situated in another (Witmer and Singer, 1998; Riva et al., 2003; Diemer et al., 2015). VR has several advantages for mental healthcare. Among other things, it can increase treatment motivation of patients because they enjoy using the technology; ensure that the content and form of interventions are tailored to the needs of individual patients; decrease treatment costs because of higher efficiency; and facilitate therapy within a specific environment that cannot be accessed from a therapist’s office (Turner and Casey, 2014; Kim et al., 2016; Botella et al., 2017). These qualities make VR an especially appealing technology for psychological treatment, since mental health problems such as phobias, alcoholism or even extreme paranoia are closely intertwined with the perceived environment (World Health Organization, 2004; Freeman et al., 2017). Reviews have indeed shown positive effects of VR interventions for mental disorders such as specific phobias (Botella et al., 2017), PTSS (Botella et al., 2015), psychoses (Veling et al., 2014), and eating disorders (Ferrer-García and Gutiérrez-Maldonado, 2012). According to several reviews, few studies focused on the use of VR with very complex and multifaceted disorders, patients and types of treatment (Turner and Casey, 2014; Freeman et al., 2017). Examples are patients suffering from multiple disorders, mental retardation, chronic psychiatric problems, and (closed) mental health settings such as hospital wards or forensic units (Freeman et al., 2017). Nevertheless, our recent review pointed out that forensic psychiatric patients – often residing in secured settings and suffering from complex disorders – might especially benefit from the immersive qualities of VR (Kip et al., 2018). More research into the ways VR can be used for these intricate mental disorders, mental health settings and types of patients is required.

Forensic mental health is a subdomain of psychiatry which deals with the assessment and treatment of in- and outpatients whose behavior has led, or could lead, to offending (Mullen, 2000). Forensic mental health has several specific characteristics. It’s main difference with regular mental healthcare is that preventing delinquent behavior is an important treatment goal, so treatment takes place at the intersection between law and psychiatry (Arboleda-Florez, 2006). Furthermore, forensic patients often have little treatment motivation, low literacy levels, and are heterogeneous in type of offense, psychopathology and risk factors, so different patients have different treatment goals (Drieschner and Boomsma, 2008; van der Veeken et al., 2018). Also, there are differences in security level: forensic outpatients live at home and receive treatment at an outpatient clinic, whereas inpatients reside in forensic hospitals while preparing for their return to society. All of this points out that forensic mental health is a setting with multiple unique characteristics. VR has been suggested by multiple authors as a potentially effective intervention strategy for forensic in- and outpatients (Renaud et al., 2010, 2014; Fromberger et al., 2014; Benbouriche et al., 2016; Kip et al. unpublished). VR can elicit emotional responses similar to those in real-life situations that are inaccessible for in- and outpatients because of security levels or ethical concerns, for example in the case of sexual offenders (Fromberger et al., 2014; Renaud et al., 2014). Furthermore, specific behavioral skills and coping strategies can be trained in controlled environments that are tailored to the individual patient’s dynamic risk factors, without endangering others (Fromberger et al., 2014). Unfortunately, there is little empirical proof to support these claims: to our knowledge, no experimental studies on the use of VR in forensic mental health have been published yet. Most published studies on VR in forensic mental health focus on the assessment of sexual offenders (e.g., Renaud et al., 2014), and even less is known about possibilities for other types of forensic patients. These gaps in knowledge cannot be filled by generalizing findings from studies on VR in other mental healthcare domains to forensic mental health because of its aforementioned unique characteristics. Especially when not much is known yet, VR applications for specific domains should be thoughtfully developed (Kim et al., 2016) to ensure that they fit the context, patients and therapists that will use it.

While a new wave of VR intervention development is approaching – or perhaps has already arrived – (Turner and Casey, 2014), very little attention has been paid to how these interventions should actually be developed and which development methods are suitable for complex domains and disorders (Dugas et al., 2017; Freeman et al., 2017). The importance of a good development process to guarantee a fit between technology, people and the context has been acknowledged by multiple studies (Coiera, 2004; Nielsen and Mathiassen, 2013; Feldman et al., 2014; Glasgow et al., 2014; Beerlage-de Jong, 2016). A sound development process should start with a thorough contextual inquiry in which stakeholders such as patients and therapists are actively involved (van Gemert-Pijnen et al., 2011). During a contextual inquiry, multiple methods are used to get a good grasp of areas of improvements of a specific context, to investigate how technology can contribute to resolving these issues, and to determine who might benefit from the technology in what way (Holtzblatt and Jones, 1993; Wentzel, 2015). Especially when there is little knowledge on the use of a technology in a specific domain, it is important to thoroughly analyze when and how a technology can be of added value. A way to do this is via participatory development, which promotes a structural cooperation with end-users and other important stakeholders via the use of multiple methods, mainly to ensure that the perspectives of these stakeholders are accounted for (van Gemert-Pijnen et al., 2011; Beerlage-de Jong, 2016). Scenario-based design is a method that fits well with participatory development. On the one hand it can be used to elicit concrete narratives from stakeholders about situations that illustrate which aspects of a current situation can be improved (Lim and Sato, 2006; Anggreeni and Voort, 2008). On the other hand, scenarios can be created by researchers to explicitly describe the hypothetical use of a to-be-developed product. These concrete scenarios can be used to support stakeholders in making their needs and preferences explicit (Beerlage-de Jong, 2016). Because methods from scenario-based design can be used to identify points of improvement and preferences regarding a technology according to stakeholders, this approach is a good first step in determining in which ways VR can be used to be of added value for forensic mental health.

In order to create a broad, multifaceted picture of the potential of VR for forensic mental health, the current study combined two scenario-based methods with stakeholders. In interviews, therapists and patients have been asked to provide scenarios themselves to gain insight into the current treatment situation and broad possibilities for VR. Based on these scenarios, concrete examples of the application of VR in forensic mental health have been be created. Scenarios that illustrate the use of these examples in treatment have been be presented to patients and therapists in an online questionnaire, in order to gain a more detailed view of their opinions, preferences and ideas for the use of VR in treatment. Via the combination of these two scenario-based methods, a broad, multifaceted picture of the possibilities that VR offers treatment in forensic mental health can be painted. This information can serve as a foundation for the development of VR applications in forensic mental health, and possibly also settings that bear similarities to forensic mental health, such as closed psychiatric hospital wards. The main goal of this paper is to identify what the added value of VR for treatment in forensic mental health can be. The three accompanying research goals are to identify (1) points of improvements in existing forensic mental health treatment of a forensic hospital with in- and outpatients, (2) possible ways of using VR that can improve current treatment, and (3) positive and negative aspects of the use of VR for the current treatment according to patients and therapists.

## Materials and Methods

In the current study, multiple methods have been used to answer the research questions. The results from two focus groups with patients and therapists were used to structure the interview scheme that was used to interview other patients and therapists. The results of these interviews served as the foundation for six scenarios on possible VR applications that were used in an online questionnaire. In this paper, the focus lies on the interview and questionnaire, of which the methods are described below.

### Study 1 – Interviews

#### Participants

Both therapist and patients were included in this study since they are important stakeholders and (potential) end-users of a future VR intervention. Therapists and patients were recruited at a forensic hospital with in- and outpatients in the east of the Netherlands. All therapists directly involved in any type of treatment were eligible to participate in this study. Convenience sampling via team leaders was used to include therapists. Eight different locations that were representative of the forensic hospital were selected, and one therapist was recruited from each location. Patients were recruited via two therapists who were part of a project team for the development of a VR application. Patients could not participate if they were diagnosed with a current psychosis or mental retardation, or if a therapist indicated that dangerous situation during the interview might arise. Participation was only allowed when a therapist indicated that the interview would not be uncomfortable or damaging for the patient. Initially, the goal was to involve eight patients, but inclusion was found to be difficult due to unwillingness to participate, so three patients were interviewed.

#### Study Procedure and Interview Scheme

The eleven interviews were conducted in May and June 2017 by one researcher (KW) and had taken place at the location of the forensic hospital that was most convenient for the participant. The interviews took between 25 and 50 min, with an average of 32 min, excluding the introduction and signing of the informed consent. All interviews were audio-recorded and transcribed verbatim.

A semi-structured interview scheme was used to elicit scenarios of treatment situations that could be improved via VR, according to therapists and patients. Throughout the interview, probing questions were asked to gain more information about classic scenario elements such as actors, their goals, the setting, activities and possible events (Rosson and Carroll, 2002). The interview started with a brief introduction. The introduction began with demographic questions and an explanation of the nature of the interview, which focused on eliciting concrete examples of situations that could be improved with VR. During this introduction, it was explicitly pointed out that the goal of the interview was not to come up with concrete ideas for the content of a VR intervention. In the first part of the interview, the participant was asked to come up with areas of improvements in current treatment, without any input or structuring from the researcher. During the second part of the interview, three categories of possible applications of VR were presented and participants were asked to describe situations in treatment where these might be beneficial. The three categories of types of VR were based on the outcomes of two previously held focus groups with, respectively, 14 patients and 23 therapists from the same forensic hospital. During these focus groups, participants were asked to come up with ideas for VR-applications for forensic mental healthcare individually and in small groups. The generated ideas were coded and categorized by one researcher (HK). The coding process of the focus group data resulted in the three main categories below, which were briefly explained in the second part of the interview to provide the participants with inspiration for their answers.

• **Skills**. The use of VR to develop or practice behavioral or cognitive skills that are useful in generic, daily-life situations (e.g., social skills or skills for daily living such as grocery shopping), or directly related to the offense (e.g., aggression regulation or saying no to drugs).
• **Treatment of mental disorder**. The use of VR to treat, prevent or support patients in dealing with mental disorders such as anxiety disorder (e.g., PTSD or phobia), psychosis (e.g., facilitate early recognition), depression (e.g., using relaxing environments), and autism (e.g., training emotion recognition).
• **Insight**. The use of VR to create insight into criminal behavior by looking at behavior from another perspective. This can be by achieved observing one’s own behavior from the perspective of another (e.g., seeing a fight between parents from the child’s perspective); providing others with the patient’s perspective (e.g., showing a significant other what a psychosis looks like); or observing how a patient reacts to a realistic situation with triggers (e.g., patient’s response to an environment with alcohol).

Two researchers, a therapist and a patient all provided feedback on the content and structure interview scheme. A pilot test was conducted with a psychologist and former patient, and minor adjustments were made accordingly. Ethical approval was given by the Ethics Committee of the University of Twente (Behavioral, Management and Social Sciences).

#### Data Analysis

Two coders independently coded all transcripts (HK and SK), using the method of constant comparison (Boeije, 2002). First, the coders read the transcripts to familiarize themselves with the content. Then, all fragments that were related to either one of the research questions were selected. We distinguished between fragments that focused on points of improvement in the current situation (research question 1) and possible application of VR that could be of added value (research question 2). Based on these fragments, two preliminary coding schemes were created inductively: codes were based on the content of the fragments and not established beforehand. We identified several main codes with accompanying sub codes and used the sub codes to code the fragments. The coders used this first version coding scheme to code the first two interviews separately. The disagreement between the two coders was discussed, and adaptations were made to the coding scheme accordingly. Both coders coded all interviews using this adapted coding scheme. After that, the outcomes of both coders were again compared. 178 Fragments were coded, and researchers agreed on 131 of these fragments and disagreed on 47. In case of disagreement, discussion took place until consensus was reached. Minor adaptations were made to the coding scheme throughout the process to ensure that the codes and their definitions optimally fit the data.

### Study 2 – Online Questionnaire

#### Participants

The target group of the online questionnaire consisted of (former) patients and therapists in forensic mental health in the Netherlands. We made use of convenience sampling and recruited participants in several ways. On a national level, the link to the questionnaire was posted on national websites, in newsletters and via a national conference on forensic mental health. Additional sampling activities were conducted in the forensic hospital in the east of the Netherlands where the interviews took place. Flyers were distributed amongst employees and the in- and outpatients, the link was posted on the website of the forensic hospital and e-mails were send to the staff.

#### Study Procedure and Questionnaire

In the scenario-based questionnaire, six examples of the use of VR in forensic mental health were presented to the participants via six videos of on average 1.5 min. These scenarios were generated by a multidisciplinary project group consisting of researchers, patients and therapists, and were based on the outcomes of the interviews. A brief explanation of the content of the six ideas is presented in Attachment 1, and the videos can be watched here: https://bit.ly/2sYkbTM. The questionnaire started with a brief introduction, an informed consent and questions on demographics and other relevant background information. After that, the videos were presented to the participants in randomized order in order to ensure that all videos would receive a comparable number of responses. After watching each video, participants were asked to grade the idea and filled in the Personal Involvement Inventory (Zaichkowsky, 1994). Since the goals of the current study are of qualitative nature these quantitative results are beyond the scope of this paper and will be discussed elsewhere (Kip et al., unpublished). After the grade and the PII, three open-ended questions were presented: one question on what participants found positive, interesting or exciting about the idea, one question on what they found negative, less appealing or unfavorable, and one on suggestions to improve the idea. In total, 108 participants, of which 19 (18%) were patients and 89 (82%) therapists, participated in the questionnaire. On average, participants spend 21 min on the questionnaire, and 49% of all participants fully completed the questionnaire. Ethical approval for this study was given by the Ethics Committee of the University of Twente (Behavioral, Management and Social Sciences).

#### Data Analysis

For the analysis, the answers to the three open-ended questions of all six scenarios were analyzed together because we were interested in positive and negative aspects of VR in general, and not specific for each idea. Two coders independently coded all answers (AK and IB), applying an inductive, iterative approach based on the method of constant comparison. Consequently, the data of the questionnaire was analyzed in the same way as the interview data. Two coding schemes were created: one on potential positive, and one on potential negative aspects of VR. Most answers to the question on suggestions could be categorized under either positive or negative aspects. The remaining suggestions were either too specific or focused on details of the ideas, so they could not be used to answer this study’s research question. Consequently, we did not provide a separate table with suggestions. The initial two coding schemes were developed by the two researchers based on the answers that were given for two ideas. After elaborate discussions with another researcher (HK), the coding schemes were adapted and used to code all data. Throughout this process, constant adaptations were made to make sure the codes fit the data as closely as possible. The same main codes were identified for the positive and negative aspects, but both coding schemes contain different sub codes.

## Results

### Study 1 – Interviews

#### Demographics

A total of 11 participants were included in this study, of which eight therapists and three patients. Three therapists were male and five were female, their average age was 46.88 (*SD* = 14.11), and their experience in forensic mental health ranged from 1 to 30 years, with an average of 14 years. Two therapists were psychologists, five were forensic nurses/socio-therapists, and one was an art therapist. Half of them worked in inpatient care, three in outpatient care, and one therapists worked in Forensic Flexible Assertive Community Treatment (ForFACT). All three included patients were male and were on average 48.67 years old (*SD* = 2.89). They had received an average of 2.83 years of forensic treatment, with a range of 1–4 years. One patient received inpatient care, the other two outpatient.

**Table 1:** Points of improvements of the current treatment according to therapists and patients (*n* = 11).

Main and sub codes		Definition of code	Codes^a^	Ther.^b^	Pat.^c^
**Characteristics of the forensic setting**					
	Return to society – emotions and cognitions	Patients can lack knowledge or are anxious to return to society	7	3 (7)	
	Return to society – skills	Patients can lack practical skills which are required for functioning in society	10	4 (10)	
	Return to society - recidivism	Patients might have a high chance on reoffending after or during treatment	9	5 (8)	1 (1)
**Patient characteristics**					
	Treatment motivation	Patients can have little motivation for their treatment	7	4 (7)	
	Patients’ low educational level	Patients might not have the cognitive skills to understand (parts of) their treatment	6	4 (6)	
	Emotion regulation	Patients can have difficulties in controlling their emotions during treatment or daily life	7	5 (7)	
**Treatment characteristics**					
	Skills training in context	Patients cannot practice skills in a real-life context	14	6 (13)	1 (1)
	Therapist’s insight in patient	Therapists do not always have enough insight into the cognitions or behavior of a patient	9	4 (8)	1 (1)
	Exposure therapy	Therapy in which the patient is exposed to fear-eliciting stimuli has practical difficulties	3	3 (3)	
	Physical activity	Little attention is paid to physical activity of patients	2	2 (2)	
	Involvement of significant others	Significant others often do not participate in treatment	3	2 (2)	1 (1)

*^a^The total number of times a code was mentioned in all interviews. ^b^The number of different therapists that mentioned a code, and (#) the total number of times the code was found in all interviews with therapists. ^c^The number of patients that mentioned a code, and (#) the total number of times the code was found in all interviews with patients.*

### Points of Improvement in Treatment

Therapists and patients provided scenarios of situations in current treatment that could be improved, without coming up with concrete solutions. The identified main and sub codes and their accompanying definitions are provided in Table 1.

#### Characteristics of the Forensic Setting

This main code is related to the unique characteristics of the forensic setting that distinguish it from most types of regular mental healthcare and can be accompanied by fairly unique issues. Preventing delinquent behavior and successfully reintegrating in society are important treatment goals, especially for patients that are excluded from society for a longer period of time. According to the participants, the transfer from a closed setting to living independently again is often a big transition, and it can be hard to fully prepare patients for this in a therapy room. First of all, inpatients might not be ***emotionally or cognitively prepared for their return to society***. Especially after several years of residing in a closed setting, changes will have occurred within society or in the patient himself. Consequently, some patients have a lack of knowledge or insight into activities that are required to function well in society, e.g., using the public transport or the internet. Also, some inpatients feel anxious about their return.

Second, inpatients can ***lack practical skills*** that are required for successful societal participation. Daily living skills can be underdeveloped because patients didn’t learn or practice these activities during their stay in a closed setting, as was explained by Participant 2:

“Someone who has been locked up for 10 years doesn’t know ‘outside’ anymore, so also doesn’t know the entire digital world. He’ll still go to the bank and wants to fill in paper forms while that doesn’t even exist anymore.”

Third, multiple participants indicated that another important skill is ***dealing with a new status as an offender***: patients can find it hard to explain their delinquent background in situations that are important for functioning well in society, such as job interviews or meeting new people. Finally, multiple participants indicated that an important issue related to forensic mental health specifically is that the ***chances for recidivism are high*** after return to society. Besides re-offending, patients can show other undesirable behavior such as drug abuse. Participant 7 explained some of these problems:

*“We do make early recognition plans: how do you recognize signals in yourself, and what can someone else do in that, and what can you do yourself? But it remains a piece of paper, it remains: I* should *do this, or* should *do that. But we all know that we are driving through a red traffic light every once in a while, and that we actually shouldn’t do that. Sometimes people do things they actually shouldn’t have done.”*

#### Patient Characteristics

This main code refers to difficulties in treatment that arise because of specific characteristics of the forensic psychiatric patient population, which was seen as complex by multiple therapists.

Participants mentioned that an important element of this complexity is a ***low motivation for treatment*** and an accompanying resistance to actively participate in all parts of their therapy. Also, according to therapists, a large share of the ***patients lack the cognitive skills*** to grasp all elements of their treatment. This might be because treatment activities or assignments require a certain level of abstract reasoning and reflecting that is too difficult, or the reasoning of a therapist is hard to understand, which is illustrated by a quote of Participant 4:

“They often don’t understand things and become angry and nervous because of that. If you explain things, it should be very brief, otherwise they don’t get it. There is much to gain there.”

Finally, therapists indicated that many forensic psychiatric patients have difficulties in ***regulating their emotions***. On the one hand, patients might be too anxious or stressed before or during treatment, while on the other hand, it might be difficult to provoke specific emotions that are required for specific types of treatment, as is explained in the quote below of Participant 1 on EMDR:

“Well, with EMDR you are working with eliciting that trauma, you want the anxiety to be as high as possible, and only then you start decentration. And with some people that doesn’t always work, it is advised against for people who don’t feel emotions, for example.”

#### Treatment Characteristics

This main code refers to issues that arise because of the nature of the therapy, which often takes place one-on-one, and in a closed setting or therapy room that does not resemble the real world. An often-mentioned sub code was ***skills training in context***. Participants mentioned that patients have to develop, practice and improve behavioral or cognitive skills that are required for their functioning in society during therapy sessions, such as social, emotion regulation, or relaxation skills. However, these types of skills can often only be practiced in a therapy setting - which requires a lot of imagination - and not in a realistic context with realistic stimuli and environments. Therapists are often restricted to discussing situations instead of actually practicing them, as was illustrated by Participant 2:

“People who keep on finding it difficult, who have been incarcerated for a long time or don’t have good social skills anyway. Then you’ll say: hey, practice! Some things are already being done with eMental Health, but I think that you cannot learn social skills from a screen: you have to experience and do.”

Another issue related to the limitations of treatment is that ***therapists do not always have as much insight*** into a patient’s mental disorder, problematic behavior or triggers of delinquent behavior as they might require for optimal treatment. This can be caused by difficulties with self-reporting instruments, the inability to observe specific, offense-related behavior in context, or social desirability during conversations with a therapist, which was explained by Participant 2:

“I do have a patient, and I’m thinking: what is this, then? You always have - especially with sexual offenders - social desirability. And in the social desirability, me and other colleagues as well are wondering: is this the patient? Or is this the patient in the social desirability that has been admitted to the clinic, and that knows: ‘I have to do this to progress in my treatment’? So in how far is someone calculating, and is someone controlling certain things?”

Furthermore, multiple therapists indicated that there are forensic psychiatric patients with anxiety disorders that require ***exposure therapy***, but this can be difficult to arrange, either because of legal restrictions which prescribe that a patient is to remain in a closed setting, or because of practical constraints which make it difficult to present the fear-eliciting stimuli or situations to a patient, e.g., in case of a fear of flying. Also, several participants mentioned that currently, little attention is paid to the ***physical activity of patients***, either during their day-to-day life in a closed setting, or during therapy sessions. Finally, another point of improvement was that ***significant others*** of patients could be more involved in their treatment, partly because of their (often) important role in the prevention of re-offending.

**Table 2:** Possibilities of VR to improve current treatment according to therapists and patients (*n* = 11).

Main and sub codes	Definition of code	Codes^a^	Ther.^d^	Pat.^c^
**Skills training with interaction**				
Training daily living skills	Development and improvement of general practical skills required for daily living	8	5 (8)	
Training social skills	Development and improvement of skills that are required for proper social interactions	14	5 (13)	1 (1)
Training emotion regulation skills	The development and improvement of skills to not give in to impulses	16	8 (15)	1 (1)
**Observing without interaction**				
Exposure to emotion-eliciting stimuli	Experiencing stimuli that elicit negative emotions and cognitions	21	6 (13)	3 (8)
Observing daily life	Observing regular, realistic daily life situations or environments	3	1 (1)	1 (2)
Observing behavior	Observing desirable or undesirable behavior from the perspective of an outsider	16	6 (14)	2 (2)
**Creating insight for others**				
Insight into reactions to triggers	Observing the reaction of a patient in an ecologically valid way	10	5 (10)	
Insight into the patient’s situation	Therapist and significant others can observe the point of view of the patient	10	5 (7)	2 (3)

*^a^The total number of times a code was mentioned in all interviews. ^b^The number of different therapists that mentioned a code, and (#) the total number of times the code was found in all interviews with therapists. ^c^The number of patients that mentioned a code, and (#) the total number of times the code was found in all interviews with patients.*

### Possibilities of VR to Improve Treatment

Besides points of improvement, therapists and patients also provided multiple scenarios of possible ways of using VR to improve the current treatment situation. The identified main and sub codes that arose from the inductive analysis of the interview and their accompanying definitions are provided in Table 2.

#### Skills Training With Interaction

This main code refers to the possibility of VR to develop, practice and improve specific skills in a realistic context, in which interaction with virtual avatars is possible. This type of interaction can be seen as a more realistic form of roleplaying during treatment because an ecologically valid context can be added to the interaction between therapist and patient. VR can be used for different types of skills. Participants pointed out that VR provides many opportunities for patients to develop and improve ***basic, practical skills that are required for daily living*** and functioning well in modern-day society. Participant 3 provided some examples of these types of skills:

“I would really like it if the people here can bear some more responsibilities and will also take those. That we can also offer them these responsibilities. And that ranges from daily living activities, to working, to going to the city, to getting up on your feet again, to searching a girlfriend again: the entire range.“

Second, participants suggested that ***social skills*** can be practiced in virtual environments. This refers to skills that are required for good and healthy social interactions that will not lead to any undesirable behavior. Third, ***emotion regulation skills*** can be trained in VR: coping skills that support the patient in not giving in to impulses when confronted with difficult, emotion-eliciting situations or stimuli. The following quote of Participant 7 explains this in the case of aggression:

“Yeah, and then for aggression, because I was talking about it a while ago with a patient who said: ‘if someone’s looking at me and that person doesn’t look away, it doesn’t even have to be an acquaintance…’. That patient really feels like: I am the boss and if the other one doesn’t look away… Then you get macho behavior and it goes wrong. I would like to be able to practice that. So regulating emotions, regulating aggression, eliciting aggression. That really adds something.”

#### Observing Without Interaction

This main code refers of using VR to facilitate the patient in the mere observation of virtual situations or environments, in which communication with another person does not play a role. An option that was mentioned, was the use of VR to ***expose forensic psychiatric patients to stimuli or situations*** that elicit negative emotions and cognitions. These stimuli or situations can be associated with phobias or anxieties, but might also be related to the offense, for example children in case of a pedophile. An example on exposure to drugs was provided by Participant 9, a patient:

“How do you respond to being exposed to drugs? Yes, a coffee shop in VR, or, let’s keep it simple, just a dealer on the street. And how does someone respond to it?”

Also, multiple participants suggested that forensic psychiatric inpatients can observe ***regular, realistic daily life situations or environments*** to get re-acquainted with society. Furthermore, VR might be used to ***observe desirable or undesirable behavior***, in order to increase the patient’s insight. Participants mentioned that patients can watch themselves from the perspective of an outsider but can also watch similar behavior displayed by another person. Patients can observe mental disorders such as schizophrenia, but also offense-related behavior, which was illustrated by Participant 1:

“There was domestic violence and he was then, he moved to another place. And then he heard the upstairs neighbor who was being, well, beat up by her partner. And then he said: ‘only then I realized what that looks like from the outside, through a window, so to speak’. So it worked really well there, so I believe that it will definitely have added value.”

#### Creating Insight for Others

This main code refers to the possibility of VR to give the patient’s therapists and significant others new insights into problematic behavior and/or mental disorders of a patient, in order to increase their understanding and to better support the patient. According to participants, VR can be used to provide the therapist with an increased ***insight into the patient’s reactions to realistic triggers*** when he or she is confronted with a stimulus or situation in an ecologically valid way. This can increase a therapists’ knowledge of a patient, which was illustrated by Participant 7:

“The one person doesn’t look away, the other one doesn’t look away, and then things start stirring up inside. Now you can talk about it, but if it actually happens you are not there. And in VR you are actually there, and you can see how someone responds and what it does to someone physically.”

**Table 3:** Potential positive aspects of the use of VR in treatment according to therapists (*n* = 89) and patients (*n* = 19).

Main and sub codes	Definition of code	Total^a^	Ther.^b^	Pat.^c^
**Treatment characteristics**				
Good way to practice	VR is a good way to train behavior in a realistic way	48 (10%)	44 (92%)	4 (8%)
Addition to treatment	VR offers new possibilities for and/or works better than current treatment	36 (8%)	33 (92%)	3 (8%)
Fit current treatment	VR can be used well within the current way of treating patients	31 (7%)	28 (90%)	3 (10%)
Input for conversation	The use of VR can lead to relevant topics for treatment	21 (5%)	21 (100%)	0 (0%)
Insight into patient’s behavior	The therapist gains new insights into the patient by observing his behavior in VR	20 (4%)	19 (95%)	1 (5%)
Practicing in a safe way	Patients can practice in VR without harming themselves or their environment	13 (3%)	13 (100%)	0 (0%)
**Patient characteristics**				
Insight into own behavior	The patient becomes more aware of his own behavior and its consequences	61 (13%)	50 (82%)	11 (18%)
Improvement future behavior	The use of VR leads to a positive change in the future behavior of the patient	23 (5%)	17 (74%)	6 (26%)
Suitable for specific target groups	VR can be used well for specific types of patients	21 (5%)	19 (90%)	2 (10%)
Insight into other’s behavior	The patient learns to better understand and interpret the behavior of others	13 (3%)	8 (62%)	5 (38%)
Support in reliving situations	VR can be used to help a patient re-experience a specific offense-related scenario	13 (3%)	11 (85%)	2 (15%)
Treatment motivation	An increase in motivation to actively participate in treatment because of the use of VR	9 (2%)	6 (67%)	3 (33%)
**Content**				
Adaptation of scenarios	The content of virtual scenarios can be adapted to the needs of an individual patient	36 (8%)	31 (86%)	5 (14%)
Adaptation of environments	The appearance of virtual environments can be adapted to the needs of an individual patient	31 (7%)	27 (87%)	4 (13%)
Realism of behavior	Behavior of and interaction between virtual people seem realistic to the user	23 (5%)	22 (96%)	1 (4%)
Adaptation of persons	The appearance of virtual people can be adapted to the needs of an individual patient	21 (5%)	17 (81%)	4 (19%)
**Practical**				
Visual realism	Environments and people in VR look similar to environments and people in real life	29 (6%)	28 (97%)	1 (3%)
New technology	VR offers a possibility to use new, innovative technology within treatment	17 (4%)	9 (53%)	8 (47%)
**Total**		466 (100%)	403 (86%)	63 (14%)

*^a^The total number of times a code was mentioned in all answers together, and (#%) the percentage of the total number of all 466 codes. ^b^The number of times a code was mentioned by a therapist, and (#%) the percentage of the total number of times the code was mentioned by therapists and patients. ^c^The number of times a code was mentioned by a patient, and (#%)the percentage of the total number of times the code was mentioned by therapists and patients.*

Furthermore, therapists and significant others can actually ***see the point of view of a patient***. VR can be used to provide a realistic experience of how it is to suffer from a mental disorder, for example a psychosis. Another way to gain insight into the point of view of a patient is to view how the patient experienced a situation in which an offense took place, as was explained by a patient, Participant 10:

“Yes I think especially loved ones, for me that’s the case. […] And she doesn’t see why I have become this way, so to say.”

### Study 2 – Online Questionnaire

#### Demographics

In total, 19 forensic psychiatric patients (2 female; mean age 41.53; *SD* = 7.37) and 89 therapists (62 female; mean age 38.79; *SD* = 12.51) working in forensic mental health participated in the questionnaire. In total, six inpatients participated, the remainder were outpatients. On average, they were treated in forensic mental healthcare for on average 7.44 years (*SD* = 7.89). Four patients were treated for aggressive delinquent behavior, six for sexual delinquent behavior, and nine patients did not indicate the main focus of their treatment. 89 therapists of 22 different Dutch forensic mental institutions participated. On average, they had 9.45 years of experience in forensic mental health (*SD* = 8.84). Several therapists worked in multiple settings: 49 participants worked with inpatients in closed settings, 61 delivered outpatient care.

**Table 4:** Potential negative aspects of the use of VR in treatment according to therapists (*n* = 89) and patients (*n* = 19).

Main and sub codes	Definition of code	Total^a^	Ther.^b^	Pat.^c^
**Treatment characteristics**				
No fit with current treatment	A VR application cannot be used within the current way of treating patients	14 (8%)	14 (100%)	0 (0%)
No new addition to current treatment	A VR application does not have any added value for the current treatment	12 (7%)	10 (83%)	2 (17%)
VR not necessary	Instead of using VR, regular, other activities can better be used to reach a goal	6 (4%)	5 (83%)	1 (17%)
**Patient characteristics**				
Unsuitable for specific target groups	VR might not be suitable for treatment of some types of patients	22 (13%)	17 (77%)	5 (23%)
Elicitation negative feelings	The use of VR causes unnecessary, non-functional negative emotions in a patient	15 (9%)	9 (60%)	6 (40%)
No effect	A VR scenario does not elicit or improve emotions, cognitions or behavior of a patient	10 (6%)	8 (80%)	2 (20%)
Dishonesty about own history	Patients do not give honest information that is necessary for using a VR application	6 (4%)	6 (100%)	0 (0%)
Dishonesty about effect	Patients are not honest about the feelings and thoughts that are elicited by a VR application	2 (1%)	1 (50%)	1 (50%)
**Content**				
Not generalizable to real life	The behavioral or cognitive skills learned in VR cannot be transferred to daily life	15 (9%)	12 (80%)	3 (20%)
No realism of behavior	The behavior of and interaction between virtual persons is not perceived as realistic	11 (7%)	8 (73%)	3 (27%)
**Practical**				
No visual realism	The visuals of a VR application do not resemble the real world enough	18 (11%)	18 (100%)	0 (0%)
Difficult to use	The use of VR in treatment is difficult for the therapist and patient	12 (7%)	6 (50%)	6 (50%)
Time to use in treatment	The use of VR within treatment takes too much time	6 (4%)	5 (83%)	1 (17%)
Costs	The development or purchase of VR technology are too expensive	6 (4%)	6 (100%)	0 (0%)
Too little options for adaptation of scenario	VR does not offer enough ways of adapting scenarios to fit an individual patient	4 (2%)	3 (75%)	1 (25%)
Too little options for adaptation of persons	VR does not offer enough ways of adapting virtual persons to fit an individual patient	3 (2%)	2 (67%)	1 (33%)
Too little options for adaptation of environments	VR does not offer enough ways of adapting environments to fit an individual patient	3 (2%)	2 (67%)	1 (33%)
Learning to use VR	Acquiring the skills to use VR will take too much time and effort from therapists	3 (2%)	2 (67%)	1 (33%)
**Total**		168 (100%)	134 (80%)	34 (20%)

*^a^The total number of times a code was mentioned in all answers together, and (#%) the percentage of the total number of all 168 codes. ^b^The number of times a code was mentioned by a therapist, and (#%) the percentage of the total number of times the code was mentioned by therapists and patients. ^c^The number of times a code was mentioned by a patient, and (#%) the percentage of the total number of times the code was mentioned by therapists and patients.*

#### Potential Positive Aspects of VR

Patients and therapists evaluated the scenarios provided in the questionnaire and wrote down aspects they found positive, interesting or exciting about the examples. The codes that resulted from these answers are provided in Table 3.

As is shown in the table, therapists accounted for 86% of the codes and patients for 14%. This ratio is comparable to the division between participants: 82% were therapists and 18% patients. The main code ***Treatment characteristics*** focuses on advantages of VR that were seen as potentially beneficial for the current treatment. The use of VR to learn new or improve specific types of offense-related behavior in a realistic way during treatment was mentioned most by the participants. Furthermore, only therapists and no patients mentioned the possibility of VR to practice behavior in a safe way, and the generation of new topics that can be discussed in treatment. Compared to the code Treatment characteristics, relatively more patients mentioned codes within the ***Patient characteristics*** category, which focuses on advantages of VR for patients. The most-mentioned advantage by all participants combined was the possibility to offer the patient new insights into his or her behavior. Also, many patients mentioned the use of VR to gain more understanding in the behavior of others, and the use of VR to increase their motivation for treatment. In the ***Content*** code, the focus lies on the composition of possible VR applications, for example the visual design and storylines. Three of the four codes addressed the possibility to adapt certain aspects of a VR application to an individual patient. It was also pointed out that realistic behavior of virtual avatars is important and beneficial. The last main code, ***Practical***, addresses advantages that are not related to content of VR or treatment, but about the characteristics of the technology and practical criteria for its use. A relatively high number of patients found the use of a new, innovative technology positive, while most therapists addressed the importance of a VR application that looks and feels realistic to the user.

#### Potential Negative Aspects of VR

Patients and therapists also wrote down points that they found negative, less appealing or unfavorable about the examples. The codes that resulted from these answers are provided in Table 4.

For the disadvantages, the percentage of codes mentioned by therapists and patients is again comparable to the ratio of patients and therapists as participants. For the main code ***Treatment characteristics***, potential disadvantages or barriers for using VR in treatment were discussed. Most of these codes were mentioned by therapists. The issue that was mentioned most often, was that a VR application might not fit or complement their current way of working. Again, relatively more patients mentioned codes belonging to the ***Patient characteristics***. The code that was identified most often was that VR might not be suitable for specific types of patients, for example patients with a current psychosis. Relatively many patients pointed out that the use of VR could cause unintended, unnecessary negative feelings, for example an abundance of anxiety because of a specific stimulus. Relatively few codes were identified for the main code ***Content***, in which possible disadvantages or pitfalls of the content of a VR application were discussed. Participants worried that skills learned via the VR application would not be relevant for real life and indicated that behaviors and conversations with virtual avatars should resemble the real world as closely as possible. A broad range of codes was identified for the last main code, ***Practical***. The most mentioned disadvantage was that the visual design of a virtual environment would not look realistic enough. This disadvantage was only mentioned by therapists, not by patients. Relatively many patients mentioned that VR might be difficult to use and that learning to use VR might take a lot of time and effort.

When looking at the tables, it becomes apparent that more positive (466) than negative (168) codes have been identified. While both tables provide a broad range of codes that differ from each other, some positive and negative codes seem to contradict each other. For example, visual realism is mentioned as an advantage, but also as a potentially negative aspect. Also, the fit with the current treatment was seen as a positive, but the lack of a good fit with the current way of working was mentioned as a disadvantage as well. Finally, a potentially positive aspect of VR was its suitability for specific groups of patients, but it’s non-suitability for specific types of patients was also seen as a barrier.

## Discussion

This study aimed to identify points of improvement of current forensic mental health treatment, to find possible ways of using VR to improve the current situation and to identify potential positive and negative aspects of VR according to therapists and patients. Several points of improvement arose from the interviews. First, being isolated from society might cause difficulties for inpatients when preparing for or actually returning to society. Second, the complex and diverse nature of the often low educated patient population was described as difficult. Third, treatment often doesn’t take place in the setting where problematic behavior is displayed, and thus a realistic context for skills training or the observation of behavior by a therapist is lacking. During the interviews, multiple ways in which VR can address these points of improvements have been identified. While the results of the questionnaire pointed out some new possibilities, most of the findings from the interviews were underlined and specified by these results. First, VR can overcome issues related to the - often closed - forensic setting by offering skills-training in and observations of realistic scenarios, which can be used to overcome practical, legal and safety barriers. Via VR, new behavior can be learned or improved. Second, VR can fit the forensic psychiatric patient population via emphasis on doing instead of abstract talking. Also, virtual persons and scenarios can be adapted to the needs of individual patients, resulting in a personalized intervention, which might also improve treatment motivation. Third, VR addresses characteristics of treatment by providing the therapists with new ways of gaining insight into a patient’s behavior and cognitions, and by letting significant others experience the point of view of a patient. With regard to potential negative aspects, therapists and patients feared that a VR application cannot be sufficiently adapted to an individual, might not be realistic enough, or that it does not have any added value for existing treatment. The answers of both methods show several contradicting codes, which partly points out the differences between the opinions and preferences of the different participants. Based on these results, it becomes apparent that there are numerous ways in which VR can be of added value for treatment in forensic mental health, as long as the opportunities technology offers are adapted to the characteristics of the patients, therapists and forensic context.

An important finding that arose from both the interviews and questionnaire, was that VR should serve as a bridge between a closed setting or therapy room, and real-life situations. The broad range of possibilities of VR to practice behavior in a realistic context and to equip offenders with cognitive and behavioral skills to prevent recidivism, without endangering others, has been pointed out by multiple authors (Benbouriche et al., 2014; Fromberger et al., 2014; Renaud et al., 2014). Many therapists indicated in the interviews that a large share of the forensic psychiatric patient population has difficulties with abstract reasoning and lacks a certain amount of imagination required for roleplaying. According to participants of the interviews and questionnaires, performing behavior in a realistic virtual scenario instead of discussing it can be of much added value for these patients. This possibility is underlined by a recent review, in which the authors stated that people can react and behave in a genuine, realistic way in VR environments (Gonzalez-Franco and Lanier, 2017). Experiencing emotions and behaving in VR as one would do in circumstances in reality is a behavioral correlate of a sense of ‘presence’ (Riva, 2011; Slater and Sanchez-Vives, 2016). This sense of presence can improve skill acquisition and knowledge transfer, partly because of a situated performance in VR (Martirosov and Kopecek, 2017). However, several participants of the questionnaire indicated that skills learned in VR might not be transferable to real life. More research is required on transfer of skills and the added value of VR compared to in-person role-playing. Nevertheless, the findings of our studies, combined with existing literature on presence in VR, endorse the potential of VR in overcoming current problems with practicing and observing behavior in context.

Another theme that often recurred in the interviews and questionnaires is related to the potential of VR to acquire new insights into patients. Current risk assessment instruments provide evidence-based ways of gaining a thorough understanding of a patient’s static and dynamic risk factors, but the accuracy of such risk assessment tools is imperfect, for example with regard to predictive validity – the extent to which the scores on these tools can predict recidivism (Douglas et al., 2017). Interviewed therapists indeed indicated that there still remains a lot that is unknown about a patient, for example about behavior outside of the therapy setting or reactions to realistic triggers such as drugs or aggressive others. Because of VR’s aforementioned qualities, participants suggested that therapists can study patient’s responses to a VR-scenario in an ecologically valid way. Via this information, therapists can gain more insight into risk factors and make their treatment even more responsive to the individual patient. Several researchers have used VR in a comparable way: they studied the use of VR to assess reactions to virtual children in pedophilic men and combined this with Penile Plethysmography (PPG) to also assess physiological arousal (Renaud et al., 2005, 2014; Benbouriche et al., 2014). Eye tracking was also suggested as a means of improving assessment of sexual offenders in VR (Fromberger et al., 2015). Based on the ideas of the interviewed participants, it seems that this way of using VR can be applied to other types of patients and offenses in an ethical sound, ecologically valid and safe way as well, e.g., to identify triggers for reactive aggression. However, there is much research that needs to be done to answer questions about reliability and validity of such approaches. Furthermore, it is important to ensure that these types of VR applications fit existing assessment and treatment approaches, such as the Risk-Needs-Responsivity model (Bonta and Andrews, 2007).

A third important finding is related to the importance of personalization of VR. Both the conducted interviews and literature show the broad nature of the forensic psychiatric patient population (van der Veeken et al., 2018). Therefore, participants also provided a broad range of areas of applications of VR, e.g., for aggression, autism, mental retardation, sexual offenders, psychosis, etcetera. This implies that a one-size-fits-all intervention is not suitable for forensic psychiatric patients (Kip et al., 2018; Kip et al., unpublished). Indeed, an important theme that came forward in the answers to the questionnaire was the importance of adapting virtual persons, environments and scenarios to the needs of individual patients. Consequently, it should be possible to personalize a VR-intervention to the characteristics of individual patients, mainly to ensure that a technology is personally relevant. Participants of the questionnaire indicated that the virtual environment, persons, and the scenarios should be personalized. This might be especially important for patients that have difficulties with abstract reasoning and imagination. Studies on personalization of eHealth in general showed that adapting an intervention to a specific individual can lead to more effectiveness (Kaptein et al., 2015; Lentferink et al., 2017). Furthermore, personalization might also lead to more treatment motivation, which was an important topic in both interviews and questionnaires, especially for patients. Based on the results of the current study, we expect that personalization of VR interventions would be beneficial for treatment, but since not much is known about the working mechanisms and benefits of personalization in VR yet, more research on this topic is required. Merely stating that personalization is important will not suffice on the long-term: once a personalized VR intervention is developed, evaluation studies should pay attention to questions about the added value of personalization, which way of using VR works best for which type of patient, and if VR can and should be used for all types of patients.

Finally, it is important to note that the results of this study do not serve as ready-to-use ideas for interventions: the identified avenues should be further developed in a systematic way, for example via the multidisciplinary CeHRes Roadmap (van Gemert-Pijnen et al., 2011).

### Strengths and Limitations

The main strength of this study is the combination of two scenario-based methods. The results of the interviews provided much insight into the current situation and could be used to create valid, realistic scenarios for the questionnaire. The findings of both methods complemented each other: the results of the questionnaires were used to further specify the results of the interviews and validate the most important conclusions. This combination of methods resulted in conclusions and recommendations with a solid foundation.

In the interviews, patients and therapists were asked about scenarios on their own experiences and ideas in an open, explorative manner. Often, stakeholders are involved as mere informants who are asked to react to ideas of researchers or designers (Scaife et al., 1997), but by applying this bottom-up approach, we gained many valuable insights into the current situation and promising directions. When used with therapists, this method resulted in a broad range of points of improvements and possibilities. No new codes were identified in the last interviews. Nevertheless, this way of interviewing appeared to be difficult because it requires a specific way of asking probing questions to elicit complete scenarios. Because several scenarios were not as elaborate as they should have been, there was some disagreement between researchers during the coding process, but after discussion, consensus was reached on all codes. While this way of interviewing seemed to be suitable for therapists, it proved to be more difficult for patients. They especially struggled with the first, broad part of the interview in which no input was provided by the researcher. We found slightly more points of improvements in the second part, in which the categories from the focus groups were used. This is in line with another study on participatory eHealth development, which stated that merely asking about needs and wishes requires a too great amount of imagination, so concrete examples should be used to prompt reactions and ideas (Beerlage-de Jong et al., 2017). Since the interviews with patients did not provide us with enough results and including patients proved to be very difficult, we decided not to continue with the interviews and initiated a complementary method: a questionnaire that made use of concrete scenarios.

While including patients in the questionnaire proved to be more difficult than therapists, the quality of the patients’ responses proved to be much higher than in the interviews. This is underlined by the fact that the percentage of codes found in the answers of patients were in line with the ratio of patients that participated. Consequently, time-consuming methods that require a certain level of abstract thinking, such as the hour-long interviews, might not be suitable for most forensic psychiatric patients, while methods that are experienced as enjoyable, such as an online questionnaire with videos, can promote more involvement in vulnerable populations (Dugas et al., 2017). Still, including patients in the questionnaire proved to be more difficult than including therapists. There might be several reasons for this. Dugas et al. (2017) state that participating in research should be rewarding for vulnerable patients. It might be that forensic patients did not perceive any direct rewards or positive consequences in participating in the interviews and questionnaire. Also, forensic patient populations are known for their low treatment motivation (Drieschner and Boomsma, 2008), which might explain the low motivation to participate in research. Unfortunately, too little research on methods that are suitable for these types of populations is available. To support researchers in choosing methods that fit vulnerable target groups with specific characteristics, for example low educational level or severe psychiatric disorders, studies should pay attention to the development process of their eHealth intervention, publish about these processes in a replicable and transparent way, and critically reflect on the used methods.

An important limitation of both studies regards the representativeness of the population. The interviews were conducted in one forensic hospital in the Netherlands, which might raise questions about the generalizability of results to other forensic hospitals. However, half of the therapists that participated in the questionnaire worked in other forensic settings in the Netherlands, which enhances the generalizability of the results. Most patients that filled in the questionnaire received treatment from the same hospital in which the interviews were held, but on average they received 7,5 years in forensic care, which makes it very plausible that a large share of these patients has experience with different types of treatment with differing levels of security (Nederlandse Zorgautoriteit, 2017; Stichting IFZ, 2017), which increases the generalizability of their opinions. Finally, while the Dutch forensic mental healthcare system differs from that of other countries, most findings of the current study seem to be consistent with recommendations of studies from other countries (e.g., Benbouriche et al., 2014; Fromberger et al., 2014), so we believe that the identified possibilities of VR are valuable for different countries as well.

## Conclusion

The results of both qualitative, scenario-based studies provide insights into the added value of VR in treatment of complex populations such as forensic in- and outpatients, and can also serve as input for new, meaningful VR-interventions that have the potential to improve quality of care, if developed and implemented thoroughly. There is not one optimal way of using VR, but a broad range of possibilities that can improve treatment in forensic mental health, e.g., developing new skills in context, exposing patients to the outside world, or providing therapists with more insight into a patient. This study pointed out that personalization is essential to make the most out of all these possibilities: VR scenarios should fit the individual needs, characteristics and treatment goals of a patient. While there is much potential, we should remain critical of when VR has added value and for whom. A thorough and continuous development and evaluation approach, in which methods that are suitable for this complex setting are used, is key for sustainable use of VR in forensic mental health.

## Author Contributions

HK, KW, DD, and YB contributed to the design and planning of the study. HK, DD, and YB collected the focus group data. KW collected the interview data. HK and DD collected the questionnaire data. HK and SK analyzed the interview data, AK, IB, and HK analyzed the questionnaire data. All authors contributed to the reporting and interpretation of the results and approved the manuscript.

## Conflict of Interest Statement

The authors declare that the research was conducted in the absence of any commercial or financial relationships that could be construed as a potential conflict of interest.

## Attachment 1 – Description of the Scenarios Used in the Questionnaire

All videos were between 1 and 2 min and all contained a brief explanation of the goal of the VR application, the embedment in the existing therapy, and an example to illustrate the idea, and an explanation of the desired outcomes. Voice-overs were added to clarify the video and provide further explanation where necessary. The ideas used in the videos are described below. All videos (audio in Dutch, but with English subtitles) can be watched via the links after the descriptions of the video.

### Triggers and Helpers

This type of VR application focuses on dealing with specific triggers, which can elicit undesirable feelings, thoughts and behaviors in patients. Examples are alcohol, fans of another football club, drug dealers or, as shown in the video, women. In this type of VR application patient and therapist can look for helpers that support the patient in dealing with triggers (https://youtu.be/cKg6M1yoSa8).

### Observing and Interpreting Body Language

This VR application focuses on the reaction of patients to the body language of another person in daily life. The patient observes situations in which the non-verbal behavior of another is central. Think of a person that walks too close by, a cashier that seems to ignore you, or someone on a terrace who stares at you. The patient discusses the thoughts and feelings that arise in these situations with the therapist, and together they look for more appropriate reactions (https://youtu.be/iBOizRz0xG8).

### Body Language and the Effect on Others

This application focuses on insight of a patient into the influence of his or her body language on another person. The influence of the environment, e.g., a quiet or crowed room, is also accounted for in this. The patient gains more insight into the effect of one’s own body language, such as an intimidating posture or restless, agitated movements (https://youtu.be/7sIKte7Dmo0).

### Roleplaying in Context

This idea for a VR application focuses on practicing social skills via a roleplay in a virtual environment. The therapist can play the other person via a voice-distorting microphone. The physical appearance and environment can be adapted to optimally fit the patient. An example is a roleplay in a train compartment, a crowded bar or a football stadium. Via this application, the patient can develop and improve social skills in a realistic context (https://youtu.be/T5njasY9YBg).

### Moments of Choice

This VR application focuses on gaining insight into the consequences of one’s own behavior. The patient can be placed in different types of virtual scenarios. During a scenario, multiple moments of choice are presented and the patient has to indicate what he or she would do and why. Based on the decision, the consequences are displayed in the virtual scenario, and this is discussed with the therapist (https://youtu.be/1wGGynUqTCM).

### Offense Script

In this VR application, patient and therapist are working with a virtual construction box to create virtual environments. They can create an individualized crime scenario that the patient can enter via VR goggles. In this environment he can analyze behavior and antecedents of behavior with the therapist, and together they can look for alternative, better behavior (https://youtu.be/ZJCJMQEnfc4).
